# Editorial: Photonics-based diagnosis and treatment of infectious and inflammatory diseases

**DOI:** 10.3389/fmicb.2023.1205032

**Published:** 2023-04-28

**Authors:** Leon G. Leanse, Weili Hong, Alessandra Nara de Souza Rastelli

**Affiliations:** ^1^Health and Sport Sciences Hub, University of Gibraltar, Gibraltar, Gibraltar; ^2^Wellman Center for Photomedicine, Harvard Medical School, Massachusetts General Hospital, Boston, MA, United States; ^3^Beijing Advanced Innovation Center for Biomedical Engineering, School of Biological Science and Medical Engineering, Beihang University, Beijing, China; ^4^Department of Restorative Dentistry, Araraquara School of Dentistry, São Paulo State University-UNESP, Araraquara, São Paulo, Brazil

**Keywords:** biophotonics, infectious diseases, diagnosis, therapeutics, photodynamic therapy, antimicrobial blue light, bacterial infection, *in vivo*

In our current “post-antibiotic era” it is now indisputable that innovative approaches are required to mitigate negative outcomes associated with untreatable infections (Hansson and Brenthel, 2022). Antimicrobial resistance is ubiquitous and is responsible for significant mortality worldwide. It is well understood that the overprescription of antibiotics has contributed to the selection of antibiotic-resistant “superbugs” that have plagued public health (Baran et al., 2023). This has occurred, in part, because of the precautionary administration of antibiotics, prior to a definitive diagnosis (Antoñanzas et al., 2022). Because numerous infection syndromes can look similar (Haddad et al., 2021), without a proper diagnosis, appropriate treatment is often delayed. Adding insult to injury, the propensity for resistance development is further perpetuated via this application of unnecessary antibiotics that induce selective pressure on commensal organisms, which can eventually transfer resistance to pathogenic strains (Crits-Christoph et al., 2022).

Frequently, antibiotics are prescribed prior to official infection diagnosis due to the lengthiness of procedures, which may take hours or days to complete (Dunbar et al., 2022) coupled with potential comorbidities (e.g., diabetes) that may require urgent treatment (Rockenschaub et al., 2020). Therefore, it is reasonable to predict that if rapid and efficient diagnostic methods become available, more patients will receive a reliable diagnosis, thus limiting the selection of antimicrobial-resistant pathogens. Even with a reliable diagnosis, with the number of pathogenic microbes that are already resistant to conventional treatment, new antimicrobial approaches are urgently needed (Hansson and Brenthel, 2022).

In recent years, there has been an influx of studies that have proposed the use of biphotonic approaches targeted at increasing the diagnostic efficiency of current gold standard approaches (Andreu et al., 2011). Furthermore, there has been a multitude of studies that have exploited the use of light, both as a replacement for current antibiotics and as an adjunct therapy (Leanse et al., 2022). Our current Research Topic “*Photonics-based diagnosis and treatment of infectious and inflammatory diseases*” published a total of eight manuscripts that are focused on the latest research into biophotonics, as it is applied to both the diagnosis and treatment of infectious and inflammatory diseases.

In a “diagnosis-focused” study by Zhang et al., the authors sought to use compound Raman microscopy (CRM) as a method of identifying bacteria and elucidating their antimicrobial susceptibility. They determined the importance of developing novel techniques for bacterial infection (and antimicrobial susceptibility testing) given that the determination using gold-standard techniques can take 3–5 days to produce results. Therefore, in their study, they produced a large set of data on bacteria as a method of identifying bacterial pathogens in urine that occur frequently during UTIs. In addition, they evaluated metabolic changes in bacteria, using Raman scattering microscopy as a method to determine bacterial response to antibiotics and as a factor for determining antimicrobial susceptibility. They found that CRM was able to provide a classification of bacteria based on conventional Gram-staining approaches and provide a determination of antimicrobial susceptibility within 3 h of testing bacteria within the urine. The authors suggested that using these Raman microscopy approaches could be a powerful diagnostic tool with strong clinical applicability.

In another similar study, Yuan et al. considered the hospital infection associated with Elizabethkingia spp., which is an emerging clinical concern characterized by multi-drug resistance and severe clinical consequences. The authors evaluated the potential of optimized single-cell Raman-DIP (coupled with deuterium probing) applied to two standard strains of Elizabethkingia spp. (ATCC 13253 and FMS-007) and 29 clinical isolates obtained from hospitals in China. The antimicrobial susceptibility test (AST) readout of Elizabethkingia spp. was determined by single-cell Raman-DIP within 4 h (only one-third of the time required by the standard broth microdilution method). The authors showed highly consistent results in comparison to the gold-standard technique (broth microdilution method). Single-cell Raman-DIP could also be directly applied to positive blood culture samples, as well as samples isolated from the blood, cerebrospinal fluids, and other body fluids, as low as CFU counting numbers of Elizabethkingia spp. The authors demonstrated the possibility of applying single-cell Raman-DIP for the clinical diagnosis of Elizabethkingia spp. In addition, the authors concluded that the single-cell Raman-DIP method could be integrated into the current clinical protocol for sepsis by halving the report time. This study also confirmed that minocycline and levofloxacin are the first-line antimicrobials for Elizabethkingia spp. infection.

In a treatment-focused study, Yin et al. provided a new approach that applied a novel photosensitizer for photodynamic therapy (PDT) against drug-resistant osteomyelitis. Osteomyelitis is an inflammatory reaction that occurs within the bone and is often caused by bacterial infection. As mentioned above, because antibiotic-resistant infections have rendered numerous microbes tolerant to conventional therapeutics, the authors proposed the use of a novel PDT approach (with or without antibiotics) to manage osteomyelitis. Their reasoning for implementing this specific approach is due to the reduced potential for resistance development, as compared to conventional antibiotics. In their study, they tested a new photosensitizer, LD4, which is a synthesized amino tertaphenylporphyrin compound that is highly soluble and has low host toxicity. They combined this with a 650 nm laser (irradiance 158 mW/cm^2^; radiant exposure: 95 J/cm^2^) as a standalone treatment of rabbit tibial osteomyelitis caused by drug-resistant *Staphylococcus aureus* and an adjunct therapy with gentamicin. They found that more than 99.9% reduction of *S. aureus* viability was achieved with PDT alone and the PDT plus antibiotic group, 5 weeks post-treatment. From a histological perspective, they found that the bone tissue was repaired in both the PDT alone and PDT plus antibiotics, with the latter presenting with more significant healing. Radiological analyses revealed that the bone defects in PDT plus antibiotic group were the least severe, and the PDT alone group was the second least severe. Treatment-wise, the antibiotic-alone group presented with the most severe bone defects, being only better than the untreated control. The authors concluded that PDT plus antibiotics may have synergistic potential in the treatment of osteomyelitis, and thus may be a promising method that may permit a reduction in the concentration of applied antibiotics.

In a related study that focused on the use of PDT, Gholami et al. searched, reviewed, and summarized published papers that considered the applications of antimicrobial PDT. The authors based their study on randomized controlled clinical studies available in different databases (PubMed, Medline, Web of Science, Scopus, and Embase) up until September 2022. They investigated the future perspectives of PDT in the dental clinical setting. The authors found several clinical studies reporting aPDT as an effective adjunctive treatment modality to reduce oral pathogenic bacterial load in periodontal and peri-implant, persistent endodontic infections, dental caries, and in fungal and viral infections. Clinical studies based on aPDT in periodontal and endodontic infections are more frequently found and described in the literature than in dental caries and fungal and viral infections. Based on these findings, the authors could demonstrate the reduction of the microorganism's load associated with these infections produced by PDT. Another important finding shown by the authors was related to the use of photosensitizers and the light parameters used. Several photosensitizers, based on synthetic or natural compounds have been used, in addition to heterogeneous light parameters that were used in different studies. Several photosensitizer types and light parameters can directly impact the efficacy of aPDT and its clinical application due to the absence of standardization among the different studies. In this way, the authors suggested systematic reviews with metanalysis to demonstrate and emphasize the evidence-based application of PDT in dentistry. The use of *in vivo* studies and high-quality randomized controlled clinical trials focusing on specific PS and irradiation parameters are needed to obtain more consistent results and better standardize the parameters used. In addition, new structural photosensitizers (not unlike LD4 described above by Yin et al.) improved by carriers and adjuvants to enhance the safety, efficacy, targeting, and cost-effectiveness can optimize the therapeutic aims and help clinicians to reach the desired antimicrobial results. The authors concluded that other aspects, such as patient satisfaction with the therapy, safety, and adverse effects, should also be considered and evaluated in future clinical studies.

In another PDT-focused study, Huang et al. focused on the description of photonics-based antibacterial strategies [antimicrobial PDT and photothermal therapy (PTT)] and their mechanisms, as well as the applications of photonics-based antibacterial strategies for the treatments of oral infectious diseases. As described by the authors, PDT involves three components: a light source (under visible or invisible wavelength), a synthetic or natural PS, and local oxygen. The main mechanisms involved Type I (reactive species of oxygen - ROS) and Type II reactions (singlet oxygen). According to the authors, PDT has emerged as a minimally invasive, low toxicity, and high selectivity technique. In dentistry, the authors described that PDT has been applied in the treatment of oral diseases such as dental caries, periodontal diseases, peri-implantitis, oral candidiasis, and endodontic infections among others. In addition, the authors also described another photonics-based antibacterial therapy called PTT. PTT is a non-invasive therapy used to combat drug-resistant bacteria and biofilms. PTT shows some advantages, such as minimal systemic toxicity, broad-spectrum antibacterial activity, and no drug resistance. For the isolated application of PTT, high-power laser irradiation and a high-dose photothermal agent (PTA) are required, which may cause tissue damage. The mechanisms of PTT are based on increased membrane permeability, bacterial protein denaturation, and irreversible bacterial destruction. Looking at the advantages of the two photonics-based antibacterial strategies, the authors described that a combination of both PDT and PTT has demonstrated potential, once this association could be more functional and minimize side effects. Heat can accelerate the release and penetration of drugs, and promote the release of ROS and some chemical reactions. Another aspect that the authors took into consideration in this review is related to the current materials for PDT and PTT. According to the authors, some points should be considered. Different PSs and PTAs require distinct parameters, especially their concentrations, light dose, and exposure time related to the light source. In addition, the oral microenvironment, such as different pH, temperature tolerance of different parts, fluidity of saliva, and gingival crevicular fluid, may exert a significant influence on these materials. Based on this review article, the authors concluded that the photonics-based antimicrobial strategies (PDT and PTT), and specific photonics-based oral materials integrated with diagnosis and treatment could be a trend and the focus of future research. It is expected that more products can be transferred into the clinical setting for the benefit of the patients.

A study by dos Anjos et al. similarly employed a light-based approach, in this case, antimicrobial blue light (aBL) for the treatment of a highly invasive *V. vulnificus* infection. It is well understood that skin and soft tissue infections (SSTIs) caused by vibrio vulnificus are extremely lethal, being associated with a 50% mortality rate. With the effectiveness of antibiotics waning, as *V. vulnificus* infection becomes more tolerant to conventional antibiotics, the authors appreciated that novel approaches may be needed to reduce mortality associated with *V. vulnificus*. Furthermore, given that the invasiveness of *V. vulnificus* is extremely severe and rapid, an approach that can be implemented rapidly may additionally reduce any associated mortalities. Therefore, the authors applied a standalone biophotonics approach, aBL, as a method of reducing mortality in a mouse model of burn infection. In their *in vitro* studies, they found *V. vulnificus* to be highly susceptible to aBL-mediated killing, with 5.17 and 4.57 log_10_ colony forming unit (CFU) reduction in viability being achieved against planktonic and biofilm states, respectively. They found all strains tested to possess different endogenous porphyrins; the principal chromophore implicated in the photodynamic effect elicited by aBL. They additionally found aBL to induce bacterial membrane permeabilization upon exposure. In their *in vivo* studies, they found that aBL could effectively and significantly reduce mortality in both male and female mice infected with a bioluminescent variant of V. vulnificus. For example, in female mice, they found that aBL exposure 30 min or 6 h after bacterial inoculation (100 mW/cm^2^; 360 J/cm^2^) resulted in 81 and 86% survival, respectively, relative to the untreated control, which only had a 28% survival. The authors also performed cytokine analyses of naïve skin tissue in male and female mice, post aBL. They found five cytokines to be upregulated in male mice (e.g., MCSF) and only one (TIMP-1) to be upregulated in female mice. The authors concluded that aBL may be an effective approach to both prevent and treat infections caused by *V. vulnificus*.

In a related study, novel research by Butement et al. assessed the use of aBL emitting urinary catheter as a method to prevent biofilm formation in *Proteus mirabilis*. Their study rationale came from the fact that catheter-associated urinary tract infection (CAUTI) is an important cause of nosocomial infection, which has been shown to be a burden to public health. CAUTI is induced by the formation of biofilm on urinary catheters that can render them “reservoirs” for infection of the urinary tract. While CAUTI can be caused by a myriad of infectious agents, the authors focused on *P. mirabilius*, given that coupled with its 10–44% incidence in CAUTI, it is especially implicated as a cause for catheter blockage. They found that in using their prototype photonic catheter, *P. mirabilius* biofilm (*LD*_90_) was inhibited at a radiant exposure of 192–345 J/cm^2^. When they increased the radiant exposure to 1,700 J/cm^2^, complete inhibition was achieved. Additionally, they found a 98% inhibition of biofilm within the catheter lumen when aBL was applied (30–50 mW/cm^2^; 324–540 J/cm^2^), which suggested to the authors that this may be both a practical and cost-effective approach for the prevention of biofilms, and by extension, catheter blockage.

Through a meta-analysis obtained from nine recently published papers, Lawrence et al. investigated the decontamination of *E. coli* using aBL. In the selected papers, different pathogenic and non-pathogenic *E. coli* strains were exposed to wavelengths ranging from 395 to 460 nm. Cochrane's software for meta-analyses (Review Manager) was used to perform five meta-analyses: one included all studies regarding the reduction of *E. coli* and four subgroup-analyses. They considered intensities, wavelengths, and exposure dose as well as serovars/pathovars reported by the authors. The authors used random effects models. A determination of the CFU was used through all studies to evaluate aBL efficacy. Blue light exposure had a significant effect on the number of viable counting of *E. coli*. However, no homogenous data was observed among the included studies. The authors also reported, among subgroups, that intensity and wavelength showed evidence of impact on *E. coli* reduction. Significant heterogeneity was also presented with respect to all serovar/pathovar subgroups among the included studies. Based on the meta-analysis, the authors concluded that there is no strong evidence for the recommendation of relevant intensities, wavelengths, and exposure doses for superficial blue light decontamination in medical or food safety contexts. There is no clear information on both inoculum preparation and study parameters used. *E. coli* may be inhibited using blue light, however, the authors suggested improvement of the protocols for future investigations. The intensity and wavelength showed to exert the clearest impact among the subgroups.

## Conclusions

In conclusion, it is evident from the studies submitted to our Research Topic that strides are being made in biophotonics, with new and rapid diagnostic treatment approaches being investigated. Similarly, new and effective light-based treatment strategies that are currently being researched have undeniably contributed to our arsenal in our fight to quell antimicrobial resistance.

## Author contributions

LL and AS wrote the manuscript. WH reviewed and commented on the manuscript prior to submission. All authors contributed to the article and approved the submitted version.
